# Utilizing an explanatory case method approach to explore alternative recruitment strategies for a longitudinal randomized clinical trial of insomnia treatment in cancer survivors amid COVID-19

**DOI:** 10.1371/journal.pone.0327806

**Published:** 2025-08-06

**Authors:** Suzanne S. Dickerson, Karen P. Larkin, Dianne Loomis, Donna Tyrpak, Misol Kwon, Youngran Cha, Heba Mohedat, Grace E. Dean

**Affiliations:** 1 School of Nursing, University at Buffalo, The State University of New York, Buffalo, New York, United States of America; 2 Division of Sleep Medicine, University of Pennsylvania Perelman School of Medicine, Philadelphia, Pennsylvania, United States of America; University of Minnesota, UNITED STATES OF AMERICA

## Abstract

COVID-19 was a barrier to meeting recruitment goals in clinical trials particularly for behavioral interventions requiring innovative and evolving strategies. This paper explores recruitment approaches prior to, during, and after the in-person recruitment pause in a longitudinal randomized controlled trial (RCT) in which cancer survivors received one of two interventions to self-manage insomnia. An explanatory case study method was used to investigate pre, during, and post COVID-19 recruitment during a longitudinal RCT. Data analysis included descriptive frequencies of enrollment approaches and outcomes obtained from the research team’s weekly documented recruitment activities, and qualitative analysis of post-recruitment focus group of clinical partner experiences within the environmental context of the clinic settings. Team analysis included data triangulation between research team’s recruitment data and clinical staff experiences, and times series analysis with explanation building with team consensus on the final product. A total of 136 heterogenous cancer survivor participants were recruited utilizing both in-person and virtual strategies with an 87.5% retention rate. Variability in success of recruitment approaches over time was demonstrated within the environmental contexts. Overall, in-person recruitment was the most effective strategy (55.1%) followed by passive strategies of print outreach and social media (36.8%). A creative and persistent research team was needed to achieve the recruitment target with a high retention rate. Recruiting in-person post COVID-19 was challenging due to clinical staff barriers. The explanatory case study method offers insight into the complex recruitment process and potential approaches that could be implemented for future public health insomnia treatment studies.

## Introduction

Recruiting participants in clinical trials poses challenges for researchers and is a primary reason for early studies’ closure in the Clinical Trial database [1]. More recently, COVID-19 pandemic caused disruptions to healthcare delivery globally, both in clinical care and in existing clinical trials with changes in working practices. [2,3]. Many research studies recruitment efforts were paused, particularly those involving behavioral or non-pharmacological interventions due to requirements for patient safety, social distancing, reduced patient visits, increased staff burden, and operational costs [4]. As a result, research teams had to pivot to ensure the safety of participants and staff, necessitating the need for new innovative alternative recruitment strategies [5]. The current paper examined recruitment approaches that provided alternatives to avoid study closure and to safely meet the recruitment target in a longitudinal randomized controlled trial (RCT) during the COVID-19 Pandemic.

The study “The Efficacy of Nurse-Delivered Brief Behavioral Treatment to Self-Manage Insomnia in Cancer Survivors” was 5-year RCT (2018–2023) whose primary aim was to determine the efficacy and durability of two intervention arms among cancer survivors with insomnia symptoms. The experimental arm of the study: Brief Behavioral Therapy for Insomnia (BBTI) was a nurse-delivered, a short-term non-pharmacological treatment for insomnia that involved multi-component behavioral strategies to address factors contributing to insomnia. The attention control arm: Healthy Eating Program (HEP) included a didactic session on how nutrition affects sleep and behavioral strategies to improve healthy eating habits. Both arms received a 45–60 minute in-person or virtual education session, with baseline surveys, daily sleep diaries (7 days) that were electronically delivered with REDCap™ software and wearing actigraphy device (7 days) followed by 1- and 2-week phone call follow-ups with additional daily sleep diaries for 7 days each. The participants were then assessed at 1-month, 3-month, and 12-month intervals with repeated measures. The published study protocol provides detailed information [6]. The goal was to determine efficacy of nurse-delivered BBTI in cancer survivors in an adequately powered RCT at a large comprehensive cancer center.

The aim of our current exploratory case study was to understand the impact of COVID-19 on recruitment and explore the success of alternative recruitment strategies in an RCT study. This approach offers insights into the strategies applied to bridging the COVID-19 gap between the research team and healthcare system.

## Methods

An explanatory case study method adopted from Yin (2008) [7] was utilized to investigate a contemporary phenomenon of participant recruitment in an RCT study as a single case within a real-life context during the evolving nature of COVID-19. This study was approved by the University at Buffalo Institutional Review Board (UBIRB STUDY00002898). IRB approval was also obtained for Aim 3: focus groups to provide feedback from staff who assisted in recruiting participants in the study that used verbal consents (ID: IRB0007275).

The case study approach enabled the research team to use pattern matching in a time series analysis with explanation building during each phase to monitor, analyze progress, and explore strategies in real-time, while adapting to the challenges posed by COVID-19 [3]. To accommodate pandemic requirements, we changed from written to verbal consent and virtual interventions with IRB approval (MOD00007451). The data collected represent the chronological strategies implemented by the research team during the pre, during and post COVID-19 periods, which ultimately led to reaching our recruitment goal. The objectives of the current study were to monitor enrollment frequencies over time to describe challenges, as well as successful adaptations in recruitment strategies (i.e., face to face, versus media-based or technological approaches and environmental contexts), based on guided study propositions:

1. What alternative strategies to recruitment/enrollment were instituted by the study team during and after the COVID-19 pause?
2. How successful were various recruitment strategies in meeting the recruitment goals?
3. What technological modifications were adapted during recruitment?
4. How did the environmental contexts (i.e., clinic location of recruitment efforts and COVID-19 barriers) influence recruiting eligible participants?

### Analytical approach

Over the course of the four-year study, the research team systematically gathered descriptive data on recruitment methods, enrollment figures, and frequencies and corresponding percentages. The data were displayed in table format to depict variations in recruitment sites, clinical environmental contexts, strategies, over time related to pre, during, and post COVID-19 timing. Graphical techniques were employed to illustrate the flow of time series data [8]. The research team met weekly to record and audit the recruitment approaches from each clinical site documented in data files and tables of participant enrollment by calendar months. Rigor was maintained in data collection and analysis by holding weekly team meetings using a systematic meeting procedure that was recorded in the meeting minutes, followed by a review of the data and by a discussion of new recruitment approaches [7]. Additionally, to address Aim 3 of the original RCT which focused on evaluating the implementation of by characterizing patient- and provider-level facilitators and barriers, virtual focus groups were conducted with clinical staff who participated in recruitment. These discussions explored facilitators and barriers impacting both the implementation process and outcomes. IRB approval was obtained for these qualitative aspects of the study as well (ID: IRB0007275). Research staff contacted relevant clinical staff by email. Study information was sent to those agreeing to participate in the focus group. Verbal consent was obtained from the clinical staff at the beginning of each focus group after questions were answered and was documented on the transcripts. Due to the COVID-19 restrictions, focus groups were accomplished near the end of the study. Thus, we obtained retrospective views from five focus groups from 10 clinic staff from 4 clinics using thematic analysis [9]. Final case analysis was a convergence of evidence triangulated from team observations, documented in data tables, flow diagrams, archival records, and focus group themes.

## Results

### Chronological strategies report

From June 2019 to October 2022, the research team enrolled a heterogenous sample of cancer survivors with insomnia symptoms from multiple clinical sites, referral sources, and outreach. Cancer survivors were identified as individuals who were ≥ 1 month after treatment (hormones and targeted therapies were acceptable) for stages I – III of either breast, prostate, colorectal or lung cancer types. Insomnia symptoms were defined as those that met the criteria by scoring above 7 on the Insomnia Severity Index (ISI) [10].

Overall, the participants (n = 136) mean age was 63.82 years [SD = 9.98] and were female (55.9%). Breast cancer comprised 45.6% of the sample, followed by prostate, colorectal and lung cancer (35.3%, 14%, 5.1%, respectively). The majority had stage I cancer (46.3%) followed by stage II (28.7%) and stage III (23.5%). Refer to Table 1. for detailed demographic and clinical characteristics.

**Table 1:** Socio-demographic characteristics of the participants (N = 136).

	N or *M*	%
**Age,** mean (SD) [range]	63.82 (9.98)	[32-82]
**Sex**		
Female	76	55.9
Male	60	44.1
**Race**		
White American, not of Hispanic origin	120	88.2
Black or African American, not of Hispanic origin	10	7.4
Asian or Pacific Islander	1	0.7
Latino or Hispanic	2	1.5
American Indian/ Alaskan Native, and others	3	2.2
**Body mass index,** mean (SD) [range]	27.87 (6.07)	[14.6-49.1]
**Marital status**		
Married	92	67.6
Single	10	7.4
Separated/ divorced	22	16.2
Widow(er)	11	8.1
**Highest level of education attainment**		
Grade school	1	0.7
High school	26	19.1
2-year college	29	21.3
4-year college	34	25.0
Graduate school	45	33.1
**Employment status**		
Working full-time	42	30.9
Working part-time	25	18.4
Home keeper	2	1.5
Unemployed, looking for work	1	0.7
Student	1	0.7
Retired	57	41.9
Unable to work	7	5.1
**Cancer type**		
Breast	62	45.6
Colorectal	19	14.0
Lung	7	5.1
Prostate	48	35.3
**Stage of disease**		
Stage I	63	46.3
Stage II	39	28.7
Stage III	32	23.5
Missing data	2	1.5%

Variables with missing: marital status (0.7%); highest level of education attainment (0.7%); employment status (0.7%); stage of disease (1.5%)

The initial recruitment target goal was 158 cancer survivors with a predicted attrition of 30%, leaving 110 participants (55 in each intervention arm) to have completed the primary endpoint, ISI at 1-month. Based upon power calculation, after 40 months recruitment, the study enrollment was complete with 136 participants with lower than anticipated attrition rate (12.5%). The CONSORT Diagram (S1 Fig) details the participant flow. Combined recruitment strategies yielded 402 potential participants of which 136 (34%) were enrolled from June 2019 to October 2022 and data collection was completed in 2023. Of the 136 participants screened, 132 were randomized into experimental or control arm, and 122 completed one month follow up (primary endpoint). The study personnel achieved a high retention rate of 87.5% by keeping the participants engaged.

#### Pre-COVID-19 recruitment strategies.

Initial pre-pandemic recruitment (June 2019 – March 2020) relied on in-person encounters in clinics (thoracic, breast, urology, colorectal, and survivorship) in a major comprehensive cancer center (CCC). After IRB approval (IRB ID: Study00002898), research staff prescreened eligible cancer survivors’ medical records and shared the list with clinic staff who approached eligible participants to determine interest. Potential participants then met with the research team and signed consents. At ancillary sites, clinic staff administered the ISI questionnaire [10]. Patients scoring above 7 were queried regarding their interest in the study and their names were forwarded to research team for more information. Informational study flyers were posted in patient exam and waiting rooms at all sites inviting potential participants to contact the research team.

#### Evolving technological adaptation during the COVID-19 pause.

The original study protocol was designed to complete survey data collection an online survey manager\) REDCap™.  During our pre-COVID-19 recruitment phase, 38 (28%) were enrolled in-person and completed the initial survey on a tablet device. Follow-up survey links were sent by email, although 13 (34%) preferred paper surveys. COVID-19 shutdown (March 2020) necessitated designing virtual recruitment and enrollment procedures to secure verbal consent and conduct virtual visits. IRB approval (IRB STUDY MOD00007451) was attained May 2020. During the height of COVID-19, precautions limited on-site access to clinical sites for the research team resulting in a recruitment pause (March 2020 to July 2020). Interested pre-COVID-19 participants were contacted for virtual enrollment (July -September 2020). Once vaccines were available and in-person protocols were instituted, participants were given a choice of in-person, phone, or videoconferencing. Participants preferred videoconferencing by 54/97 (60%). Nineteen participants (19.5%) chose in-person with precautions, and one (1%) by phone. Some participants preferred their initial meeting in-person and follow up by virtual (n = 6) or phone (n = 4). Ten participants preferred paper daily sleep diaries (10.3%) and one preferred all paper surveys.

#### End of COVID-19 pause and continuing need for alternative approaches.

Emerging from COVID-19 (Oct 2020), the research team was granted access to CCC but denied clinic access until December 2020. Additionally, new strategies were required due to a recruitment lag (March 2020 – February 2021) and loss of research team personnel. The research team sought alternative strategies using resources at the Clinical and Translational Science Awards Program including: 1) *ResearchMatch* registry, 2) *Research Registry*, a centralized database of individuals interested in research; 3) *TriNetX*, a health research network access to real-time clinical data from EMRs, requiring detailed permissions, programming, and physician permission to contact their patients.

The research team hired and onboarded three new research team personnel to resume the in-person weekly screening of the medical records for eligibility in the clinics; however, recruitment was slow. Additionally, to expand recruitment to community participants, a doctoral student was hired as study personnel (0.2 full time equivalent [FTE]) for six months to recruit from an urban family practice site, libraries, and community specialty practices with minimal results. In July 2021, as part of a research practicum three Bachelor of Science Nursing (BSN) students provided approximately 0.2 FTE hours of in-person CCC clinic recruitment. The research team, with IRB approval, expanded outreach to the CCC Patient Resource Center, contacted support groups, patient advocate groups, and clinical and academic social media sites (i.e., Newsletter, Facebook, and Blogs).

#### Health newsprint sources and outreach.

Recognizing news outlets were a primary information source for the public during COVID-19, the research team published the study information in major newspapers’ weekly health supplements and a regional monthly health publication with positive results. This outreach extended the study to a broader geographic area. Community recruitment efforts also involved information tables at three outdoor community CCC hosted events. However, attendance was limited due to COVID-19 related hesitancy.

#### Cumulative recruitment by the number of participants.

Overall recruitment was illustrated using a run chart that delineated timeline of enrollment flow across time [8] (see S2 Fig) Early in the study, the initial spike in June 2019 reflected the clinical partners initial enthusiasm and referrals gathered prior to the study launch. There are two negative trends when enrollment ceased during COVID-19 pause (February 2020 – July 2020) and was slow to return (August 2020-February 2021) and four notable increases (September 2019; March 2021; October 2021 and – July 2022) showing effectiveness of several new concurrent strategies implemented (See S2 Fig).

#### Recruitment strategies summary.

Active in-person recruitment was the most effective strategy overall (n = 75/136, 55.1%). In-person approaches included the collaboration between the CCC and BSN research students yielding 5 enrollments. Due to limitations in in-person access during and after COVID-19, in-person recruitment continued to yield less recruitment overtime and the need for alternative strategies remained. The research team adding a print outreach including study flyers, newsprint and newsletters on social media strategies resulting in successful recruitment numbers n = 50/136, (36.8%). Research registries provided very few participants. Table 2 summarizes strategies, timing, and percentages per recruitment site. S3 Fig demonstrates chronological flow and variations across time.

**Table 2:** Recruitment strategies utilized to enroll 136 participants during the study enrollment period.

Method	Strategy used	Description	Site	Timing	Number (%)
**In-person**	Clinical staff referrals with research staff follow-up	● Research staff pre-screen schedules, notify staff, and approach patients about the study.	CCC: Survivorship and breast clinic, thoracic, colorectal and urology	^a^ Pre-pandemic	30 (22%)
Total n = 75 (55,.10%)		● Requiring research staff approach eligible participants significant buy-in from staff.		^b^ Pandemic	10 (7.3%)
				^c^ Emerging from pandemic	20 (14.7%)
	ISI administration	● ISI was given to each potential participant and ISI screening forms returned to research staff. Participant indicated whether research staff could contact them.	Outpatient private oncology practice	^a^ Pre-pandemic	7 (5.1%)
				^c^ Emerging from pandemic	3 (2.2%)
	Nursing student recruitment	● Research practicum where students recruited in clinics and determining interested patients who were followed up by research staff.	CCC	^c^ Emerging from pandemic	5 (3.6%)
**Print outreach and social media**	Study flyers	● Placed in exam rooms and posted in clinics and x-ray suites.	CCC and outpatient private oncology practice, libraries and CTSI	^a^ Pre-pandemic	1 (.07%)
				^b^ Pandemic	1 (.07%)
				^c^ Emerging from pandemic	16 (11.8%)
total n = 50 (36.80%)	Newsprint	● Interview with PI with study description in in local newspapers. Two subsequent briefs stating recruitment needs.	Study cite Health News	^c^ Emerging from the pandemic	29 (21%)
	Institutional social media	● Study summary asking for participants	CCC Institutional Newsletter and social media platform	^c^ Emerging from pandemic	3 (2.2%)
**Research registries**	TriNetX^d^	● Dataset limited to inclusion and exclusion criteria. Potential participants contacted via mail after primary care provider approval.	Research registry proprietary database	^c^ Emerging from pandemic	2 (1%)
Total n = 2 (1.5%)					
**Community engagement**	Community events	● Informational table at community events with recruitment staff discussing the study.	3 CCC events	^c^ Emerging from pandemic	4 (2.9%)
total n = 9 (6.5%)	Support groups and personal referrals	● Flyers given to coordinators	Breast and prostate cancer support groups	^c^ Emerging from pandemic	5 (3.6%)

*Abbreviations*: CCC = comprehensive cancer center; CTSI = Clinical and Translational Science Institute; ISI = Insomnia severity index; PI = principal investigator. *aPre-pandemic: June 2019 – March 2020; bPandemic: March 2020 – September 2020; cEmerging from the pandemic: September 2020 – October 2022; dTriNetX: Global health research network*

### Focus group evaluation of program implementation

A series of five virtual focus groups of (1–3) participants with a duration of 20–45 minutes were held June 2023-October 2023 (IRB STUDY0007275) to obtain feedback from a purposive sample from 10 clinical staff (2 MD and 8 nurses) who participated in recruitment in the study. An expert (SD) in qualitative methods virtually conducted the virtual focus groups. Participants gave IRB verbal consent to the recording of discussion at the beginning of the meeting and were reminded of the nature of focus groups related to keeping confidential others information. Participants were asked (n = 1–3) to describe the barriers and facilitators for recruitment in their settings. Transcripts were de-identified and provided 50 pages of data for thematic analysis. The research team of 4 individuals trained in qualitative analysis coded individually and met as team to discuss emergent themes of the interview transcripts until reaching saturation. Themes emerged from the data. The team identified key themes reflecting recruitment barriers at the patient, provider, and clinic levels, highlighting time-constraints during clinic visits, with staff prioritizing focus on the patients’ cancer disease outcomes versus insomnia symptom burdens. Low staffing exacerbated issues with clinic staff workload. Facilitators for recruitment themes identified the helpfulness of research staff attending the clinic and flagging eligible patients; onsite research team availability during visits to address patients’ questions; and organizing informational lunches to keep clinic staff informed about the study. In the later stages of recruitment, research team delivered flyers to clinic staff with eligible patients named on each flyer as a cue for specialty clinic nurses prior to the visit. Table 3 delineated themes and related quotes.

**Table 3:** Focus group themes and subthemes.

Themes	Subthemes	Quotes
**Identifying barriers to recruitment**	*Patient-level barrier*	
	Focusing on cancer verses sleep issues	“The survivorship people want to be quick in and out… they’re just in a different stage of their treatment and care plan….So, a barrier is really trying to help patients see the benefits of doing it, and really just getting people to agree to do it.” (P7)
	Anxious waiting	“A lot of them [patients] are very anxious if they’re follow-up patients.” (P2)
		“It is important to make sure people are getting decent sleep because they’re going through so much stress right now.” (P6)
	*Provider-level barrier*	
	Time constraints	“I would often run behind on my time schedule.” (P2)
		“It’s just that the nurses are stretched doing many things right now.” (P4)
		“Adding another task to their daily work.” (P5)
	Heavy workload	“Clinics are just busy. So, when patients start asking questions that nurses don’t have the answers to it sort of just frustrates everybody, and then they want to forget about it.” (P7)
		“We try our best to integrate everything that we can to educate the patients …then saying, “I forgot to ask about their sleep.” (P8)
	Forgetting to mention study	“Just, remembering to take the time to talk to the patients…. and just getting the staff to all remember to do it,” (P1)
		“It seems like an easy question... ‘do you have trouble sleeping?’, and then if they say yes,…we have to go into more detail and, while on the back of my mind everything you have to do, I’ve got 2 more teaches [patient instruction] … giving them all the information I need to be giving them, to see if they would be interested [in the study]. Just time.” (P8)
	Lack of awareness and buy-in with	“Buy-in is really important for the staff to understand the purpose of it. And the importance of it, and some people are just a little less willing to buy-in to doing something more than what they currently are doing in their job.” (P1)
	*Clinical-level barrier*	
	Pandemic-related barriers	“Our clinic [survivorship] has more time to do all these surveys because my boss is a researcher…we do a lot of surveys in our clinic. So that wasn’t the issue, I think. probably the follow-up, because, then COVID happened.” (P4)
	Timing of visits	The pattern of scheduling patients visits, in the survivorship clinic, they see patients yearly and the study as separate from their visits….survivorship patients really only follow with us, every 6 months or every year.” (P7)
	Lack of regular sleep screening	“Because we’re in GI [gastrointestinal clinic], we just focus on GI questions and then those mandatory questions that we have to about abuse and stuff like that.” (P10)
		“They [patients] are more focused on the particular cancer site [at other clinics], where here in survivorship, we look at all their issues. …that’s why we’re able to identify sleep problems little bit more than other clinics.” (P3)
	Study design related barrier	“I think the hardest part about these surveys is especially in a clinic. We do have several studies going at once, and it is difficult for the clinicians to stop and say, this person would meet this study.” (P4)
		“They [nurses] felt like it was a lot of like extra steps to get them [patients] to like to sign up….the only barrier is really just talking to the patients.” (P5)
		“Some [patients] have looked at the things that they needed to do for the sleep study and said, ‘I’m overwhelmed right now as it is. I don’t know if I can handle one more thing right now.’ That’s a lot of the feeling that I got from some of the patients.” (P8)
**Facilitating recruitment**	Physical presence of research staff	“The frequency of seeing the [research] staff coming by the clinic was helping to know there was engagement from the research staff for the patients…Your research staff were the persons that flagged the people that would qualify. And that was awesome.” (P10)
	Prescreening patients	“What helped the most in urology [was] when research staff started going through the schedules and identifying the [eligible] patients, which took just the load off of the nurses. We had the flyer [with the name and schedule time on it] and everything ready to go for the patient, so they didn’t forget anything, that was helpful.” (P5)
		“The [study information] sheets were in our folder for the day. We just picked it up with the patient’s information”. (P9)
		“When research staff would identify the patients, we needed to speak with, I would definitely talk to them about it.” (P6)
		“Ask them [patients] if they had any trouble sleeping…some of these people newly diagnosed or former patients, and then sleep is important for your health. So, that was important to know if they had trouble sleeping.” (P9)
	Assessing for sleep problems regularly	“We had patients who said ‘yes, I’m having trouble sleeping’ and I told them about the study, would they be interested, if they said ‘yes’. while they were there in clinic, I would let [research staff] know.” (P8)
		“[Prior to each visit] We [survivorship] did the EORTC [European Organization for Research and Treatment of Cancer, questionnaire] and we were prompted with the fatigue section, or problems with sleeping as a screen, then the nurse, if they prompted yes, would give them the study information… So, we do a lot of surveys in our clinic.” (P4)
	Informational materials and staff lunches	“We had little flyers up on in each of the examining rooms, and you know it was usually the nurses that put the patient in the room. That asked if they would have an interest.” (P3)
		“I really feel like we were informed quite a bit on what the study was about.” (P3)
**Suggestions**	Prior visit screening	“In our clinic [survivorship] our patients, are usually called a couple of days prior to their appointments, we ask them to do the quality-of-life survey which does have the sleep questions on... If they address anything through the portal prior to their visit…. You’d be able to identify at the time of the clinic visit.” (P3)
		“The better way to identify the patients or talk to the patients would be upon, their initial intake process where it just becomes a part of the workflow.” (P1)
		“We could add another question about sleep.” (P6)

## Discussion

The purpose of this study was to explore recruitment approaches during COVID-19 identifying alternative strategies overtime to successfully meet recruitment goals, institute technological modifications, and recognize influence of environmental contexts.

### Strategies

Initially, our research team did not meet the goal of 4 participants per month due to the time-intensive prescreening of eligible participants recruitment strategy at the CCC. When in-person recruitment was possible pre- and after COVID-19, recruitment required the research team’s time totaling one FTE per week. Screening medical records for insomnia required a more nuanced review and interview between the research team and the potential participants, thus nurse practitioners were the most effective research team member for screening. To address the issues of identifying potential participants who have insomnia, the research team members and clinical respondents suggested screening for sleep problems at the CCC by including a pre-appointment screening questionnaire (including problems with sleep) for patients as part of the appointment intake process [11–14]. However, the CCC would not facilitate the pre-visit assessment and clinic staff were overloaded often forgetting to do sleep assessments. Thus, recruitment approaches were needed that provided direct contact with potential participants to ask the question “are you having trouble with your sleep?” A number of studies [11,15,16] suggest a variety of recruitment strategies to mitigate low enrollment; however, COVID-19 slowdown created barriers to recruitment due to 8-month shutdowns and subsequent hesitancies. The research team found using print outreach including flyers and social media although health news sources to be particularly effective during the down time to broaden outreach to the community in general to those suffering from sleep issues. We also suggest employing community partnerships and assigning representative recruiters to assist in recruiting a broader representation of participants of the population. The teams’ continuous monitoring of recruitment and enrollment goals through ongoing data collection for this case study, facilitated a critical appraisal of strategies which facilitated reaching our enrollment goal.

### Technological adaptations

Before COVID-19, participants preferred in-person interactions followed by email survey queries, but after COVID-19 there as a necessity to shift totally to virtual interactions. When in-person protocols were reinstated after vaccines became available, participants were given the choice of in-person or virtual with 60% opting for virtual interactions. These virtual in-person approaches allowed participants to still establish trust with the project manager, mirroring the importance of initial in-person communications promoting year-long study retention. Additionally, virtual outreach through health newspapers extended our geographic reach to potential participants who recognized their need for sleep interventions.

### Environmental influences on recruitment

Identifiable issues emerging from the focus groups analysis focused on the struggle to keep CCC clinical staff committed to the recruitment process while recognizing their challenges in addressing ongoing patient concerns. Clinical staff prioritized concerns with disease symptoms, thus patient’s questions related to sleep were neglected. Although clinical providers voiced interest in helping recruit participants, they seldom mentioned the study to their patients due to other competing priorities. Time constraints of a busy clinic setting made their commitment to study recruitment less of a priority. For these reasons, it was imperative for research team personnel to be physically present in the clinic setting during the recruitment process. Potential participants from the survivorship clinic who had completed treatment had the advantage of having a prescreening quality of life survey that could identify sleep issues and were more willing to participate in clinical trials to mitigate troubling quality of life issues. After the COVID-19 lockdown ended and in-person visits resumed, many patients transitioned to telehealth clinic visits where the research team did not have screening access. Furthermore, themes from the focus groups clinicians reported that patients were worried about recurrence and waiting on scan results and did not prioritize insomnia. Some clinicians reported tending to refer patient with sleep problems to their primary care provider or to other support services such as acupuncture. Furthermore, suggesting sleep referrals may assist patients in need.

## Summary insights

The strength of this exploratory case study on recruitment practices was the insider perspective of the research team who met weekly to discuss recruitment status and brainstorm novel strategies in the context of the changes that occurred during the COVID-19 pandemic. We noted that in-person recruitment at a CCC was a strength in obtaining access to recruitment of heterogeneous cancer types. An added advantage of the CCC site was having survivorship and specialty clinics that follow patients over time versus patients who return to primary care providers.

An additional strength in our study was in the expertise of our research team, who were nurse practitioners with extensive experience in caring for cancer survivors within this healthcare system and environment. They were familiar with the clinic’s providers and proficient in navigating the medical record or screening eligible individuals. To enhance patient awareness of the study, several strategies were employed, including distributing informational flyers to clinic staff to hand to eligible patients upon placement in examination rooms.

COVID-19 lockdown inhibited our in-person contact with potential participants, which continued for 8 months. We noted the surprisingly positive influence of the informational articles about the study in the newspaper health supplement (print and digital versions) that resulted in multiple spikes in recruitment. Perhaps newsprint was a preferred information source during COVID-19. People who are interested in their health and well-being read about the study and self-initiated contact with study personnel to obtain more information. We can infer that perhaps this approach to reach cancer survivors and captures the attention of persons promoting their own self-care. Future studies of cancer survivors’ health seeking may enlighten us further on using this approach with this population. Promoting sleep health is essential to overall health especially for cancer survivors.

### Limitations and future directions

Despite the strengths and valuable insights that the current study provides, there are several limitations that should be considered. First, despite efforts to obtain a diverse sample of cancer survivors, the participants in our study were primarily non-Hispanic White individuals from a targeted geographic area (Western New York), which may limit the generalizability of the findings. However, we have provided detailed descriptions of the real-life context in which this study was conducted – specifically, the conditions imposed by the COVID-19 pandemic and related shutdown measures. Including such contextual information may enhance the transferability of our findings, as it allows readers and researchers to understand and adapt the intervention approach and results to similar real-world scenarios. Additionally, the participants were largely skewed towards either breast or prostate cancer types and may not accurately reflect the views and experiences of individuals from a heterogeneous sample of cancer survivors. During recruitment at the survivorship clinic, there was a high interest by survivors with gynecology and head-and-neck cancer types. Interest by these groups should be further explored for future trials. Second, the reasons that eligible participants declined to take part by recruitment strategy were not formally collected, which would have further aided analysis of the approach. Third, we found that some participants hesitated in joining in the study upon learning about the two arms in the study. Future research should also consider providing an option for cross over design for those randomized to the control arm after the study is complete.

To address validity threats in this case study analysis, we employed rigor by using a systematic procedure in a time series data collection approach that explicated recruitment outcomes with documentary evidence (i.e., using the consent dates). Further, we operationalized recruitment measures with flow charts, timelines and graphics in chronological order [7]. In evaluating data, the team searched for patterns in the data that predicted outcomes, identified problems, and tested solutions. These patterns provided answers to the propositions that explained the alternative recruitment processes with documentary evidence of when,where, and who was enrolled in the context of the COVID-19 pause.

During this RCT, the consent dates provided documentary evidence of the recruitment process challenges in a real-life context that was highly pertinent to the phenomenon. In team meetings we reviewed recruitment patterns that offered potential explanations of the effect of various approaches, answered initial propositions, and brainstormed new ideas. Data gathered systematically regarding different approaches provided a chain of evidence [7] to explain the usefulness of each approach facilitating analyzing the initial propositions. Furthermore, having multiple sources of evidence, such as, including the voices of clinic staff in focus groups, assisted in data triangulation to understand the chronological influence of the COVID-19 shut down on recruitment.

Lastly, rebuilding trust with the partnering clinics and providing them with continual feedback and updates during the height of COVID-19 were definite challenges. Numerous turnovers in providers, clinical staff members, and changes in rules/regulations at the partnering clinics coupled with limited in-person interaction made our study difficult to promote. For instance, most stage I cancer survivors were pivoted to telehealth visits or were referred back to their primary providers. Internally there were some changes in the research team resulting in a reduction in the number of personnel which slowed recruitment. The research team in our study consisted of former health care providers (i.e., nurse practitioners) translating to higher personnel costs. Focus group interviews with study collaborators clearly identified barriers of workload and cancer focus during clinic visits. They also suggested adding sleep assessment questions to preclinic visit assessments to sensitize patients to their sleep health behaviors and the role of good sleep in improving their health in the future.

## Supporting information

S1 Fig The CONSORT diagram. Fig 1 legend. *Abbreviation*: BBTI = Brief behavioral treatment for insomnia; HEP = healthy eating program; ISI = Insomnia Severity Index; OSA = obstructive sleep apnea. ^a^In-person: Comprehensive cancer center and screening of ISI by community partner; ^b^Other: Newsprint, social media, community flyer, research registries, etc.
(TIF)

S2 Fig Run chart of the total participants recruited over the duration of the study recruitment period (N = 136). (TIF)

S3 Fig Number of participants recruited per month using strategies employed. (TIF)
