# Supplementary figures and images for: Utilizing an explanatory case method approach to explore alternative recruitment strategies for a longitudinal randomized clinical trial of insomnia treatment in cancer survivors amid COVID-19
Source: PLoS One. 2025 Aug 6;20(8):e0327806. doi: 10.1371/journal.pone.0327806 (PMC12327650; doi:10.1371/journal.pone.0327806)

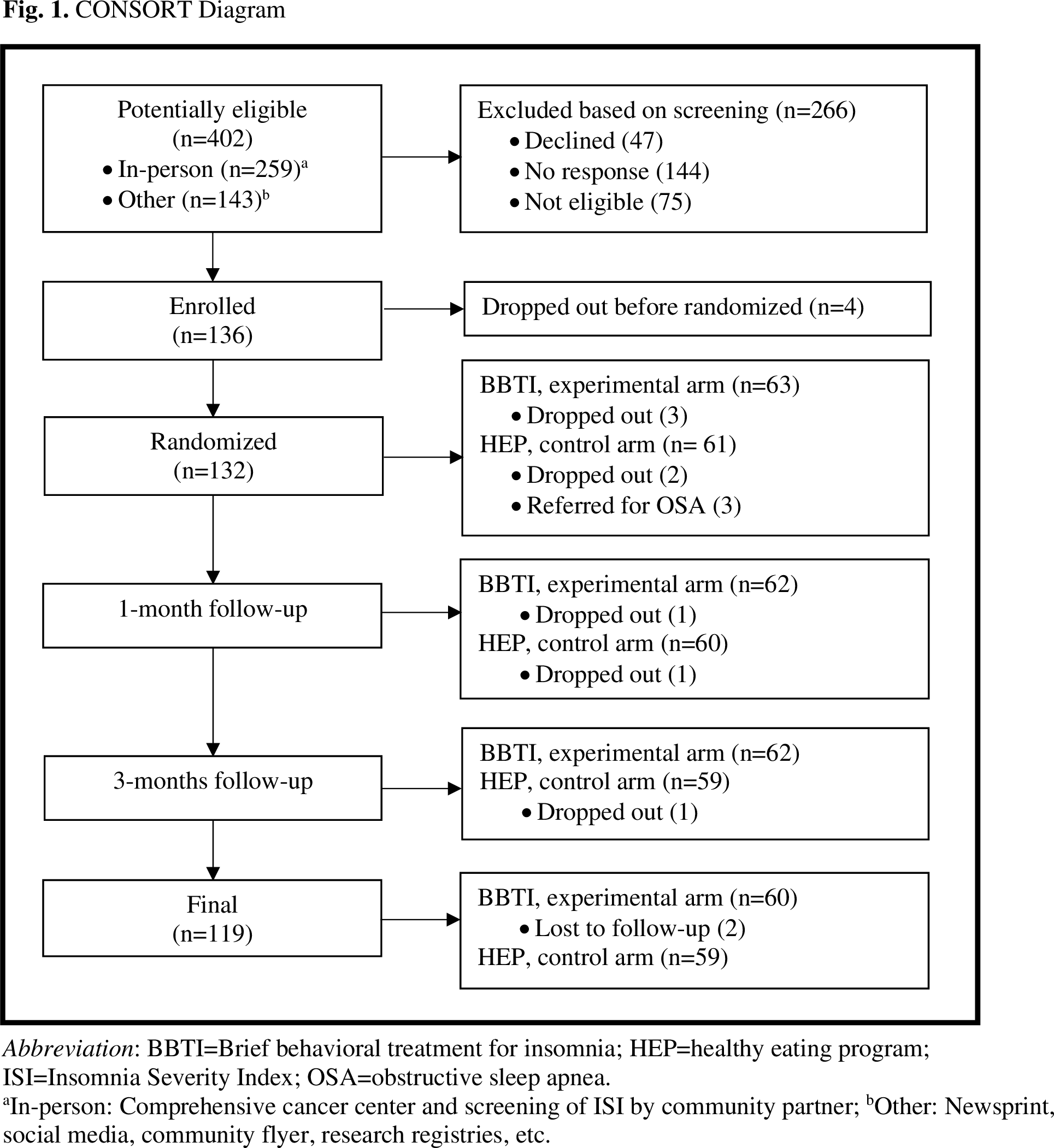

Supplement: S1 Fig — Fig 1 legend. Abbreviation: BBTI = Brief behavioral treatment for insomnia; HEP = healthy eating program; ISI = Insomnia Severity Index; OSA = obstructive sleep apnea. aIn-person: Comprehensive cancer center and screening of ISI by community partner; bOther: Newsprint, social media, community flyer, research registries, etc. (TIF) [file pone.0327806.s001.tif]

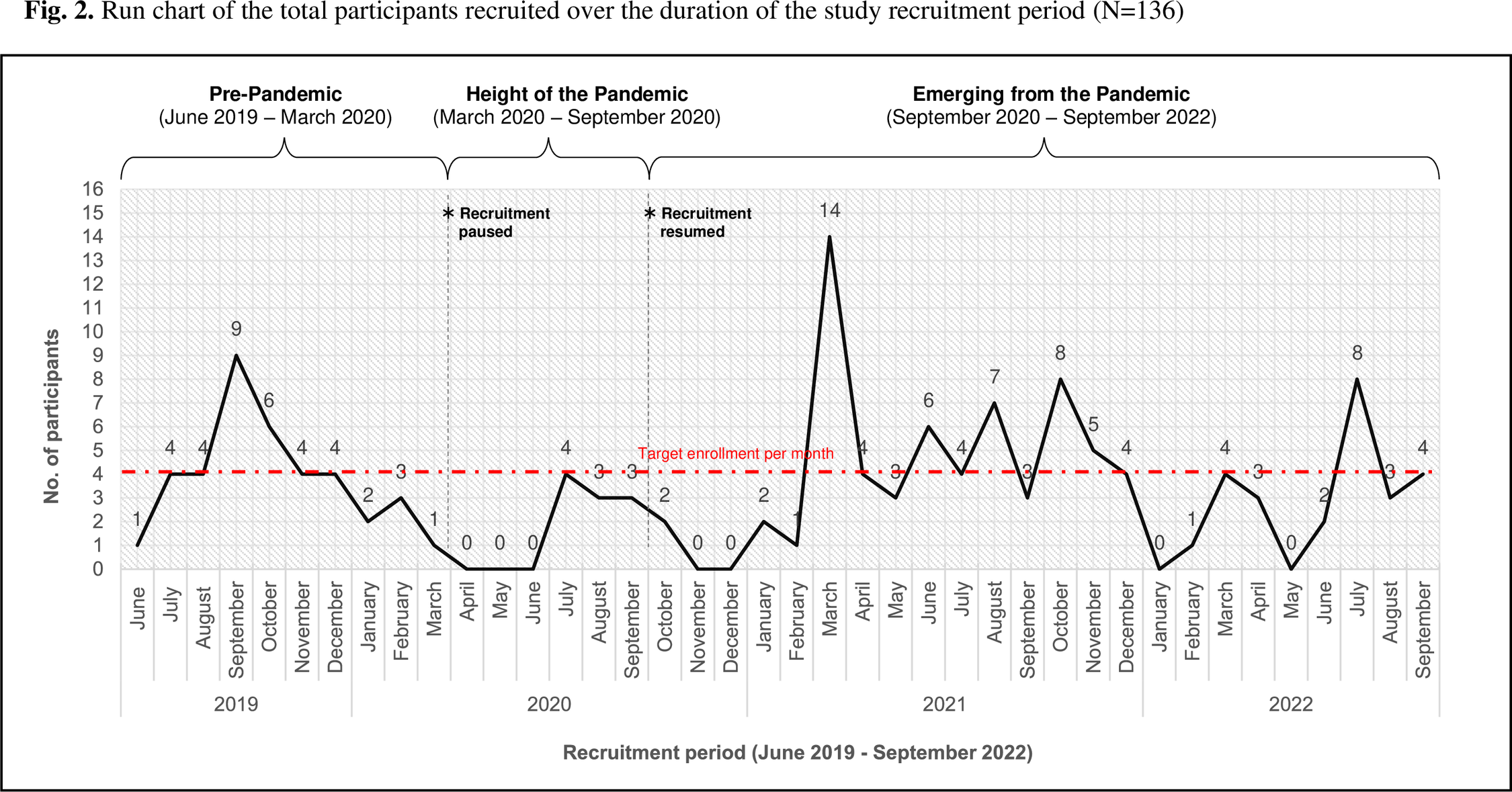

Supplement: S2 Fig — (TIF) [file pone.0327806.s002.tif]

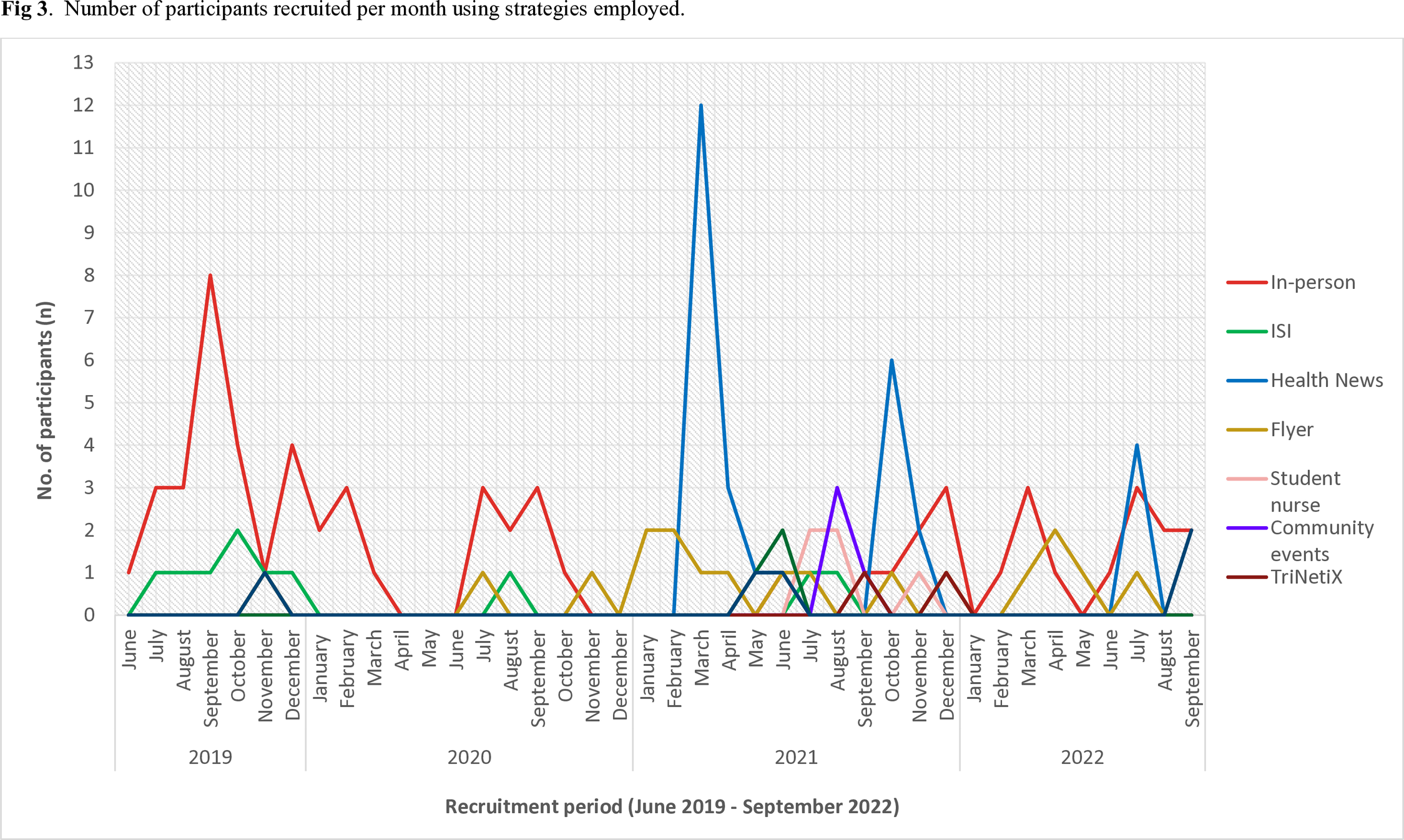

Supplement: S3 Fig — (TIF) [file pone.0327806.s003.tif]
